# Selection and Optimization of an Innovative Polysaccharide-Based Carrier to Improve Anthocyanins Stability in Purple Corn Cob Extracts

**DOI:** 10.3390/antiox11050916

**Published:** 2022-05-06

**Authors:** Lucia Ferron, Chiara Milanese, Raffaella Colombo, Raffaele Pugliese, Adele Papetti

**Affiliations:** 1 Drug Sciences Department, University of Pavia, Viale Taramelli 12, 27100 Pavia, Italy; lucia.ferron01@universitadipavia.it (L.F.); raffaella.colombo@unipv.it (R.C.); 2 FlaNat Research Italia Srl, Via Giuseppe di Vittorio 1, Rho, 20017 Milan, Italy; 3 Consorzio Interuniversitario per i Sistemi a Grande Interfase & Department of Chemistry, Physical Chemistry Section, University of Pavia, Viale Taramelli 16, 27100 Pavia, Italy; chiara.milanese@unipv.it; 4 NeMO Lab, ASST Grande Ospedale Metropolitano Niguarda, 20162 Milan, Italy; raffaele.pugliese@nemolab.it

**Keywords:** anthocyanin, alcohol-insoluble polysaccharide, by-product, camelina cake, purple corn, carrier, solid-state stability

## Abstract

The extraction process of alcohol-insoluble polysaccharides from exhausted Moradyn cob (*Zea mays* L. cv. Moradyn) (EMCP), camelina cake (*Camelina sativa* L. Crantz) (CCP), and common bean seeds (*Phaseolus vulgaris* L.) (CBP) was investigated and optimized by Response Surface Methodology. Each fraction was tested at different core/carrier ratios in the encapsulation of Moradyn cob extract (MCE), a rich source of antioxidant anthocyanins, and the obtained ingredients were screened for their encapsulation efficiency (EE%) and extraction process sustainability. The ingredients containing 50% and 75% CCP had EE% higher than 60% and 80%, respectively, and were selected for further studies. Preliminary structural analysis indicated CCP was mostly composed of neutral polysaccharides and proteins in a random-coiled conformation, which was also unchanged in the ingredients. CCP-stabilizing properties were tested, applying an innovative stress testing protocol. CCP strongly improved MCE anthocyanins solid-state stability (25 °C, 30% RH), and therefore it could be an innovative anthocyanins carrier system.

## 1. Introduction

Nowadays, there is a great concern about the consumption of foods rich in bioactive compounds, which claim not only to have a nutritional function, but also to improve health condition, thus reducing the typical chronic disease risk factors [1]. Anthocyanins represent a class of compounds deeply investigated for their several healthy properties, especially antioxidant, anti-inflammatory, and hypoglycemic [2], and use as food colorant [3]. Several papers reported that supplementation with exogenous antioxidants such as anthocyanins coming from diet or food supplements may help to reduce oxidative stress and inflammation, also preventing the obesity-related complications risk [2].

Among anthocyanin-rich foods, purple corn represents an interesting, cost-effective source of antioxidant compounds thank to the high anthocyanin concentration both in the aleurone layer and cob; the latter is reported to contain especially high amount of anthocyanins, including cyanidin-3-*O*-glucoside, pelagonidin-3-*O*-glucoside, and peonidin-3-*O*-glucoside; additionally, protocatechuic, hydroxybenzoic, vanillic, caffeic, *p*-coumaric, and ferulic acid, the majority of phenolic compounds, are also found in cob, thus representing the most interesting source of nutraceuticals to be used for the development of value-added food and food supplement products [3].

However, the main limitation of the application of such bioactive compounds consists of the easy degradation that can rapidly occur during food or food supplement processing and/or storage practice, thus strongly affecting the antioxidant potency of such compounds. In order to overcome this issue and to improve the stability and shelf life of anthocyanins, several authors evaluated the possibility of encapsulating anthocyanin-rich extracts isolated by different matrices [4]. Encapsulation is a technique that basically consists of coating a bioactive sensitive substance with a carrier (or wall material) in order to form a delivery system able to enhance the stability of the active substance during production, and preserve its antioxidant potency during storage, and possibly also passage through the gastric tract [5].

Furthermore, considering the growing demands for functional food with higher nutritional value and the growing consensus towards the use of vegetarian and sustainable ingredients, the new research goal in food industry is the application of innovative carriers, such as oils, lipids, proteins or polysaccharides, isolated from vegetable sources, mainly agro-food by-products [5].

A successful delivery system should ensure high retention of the core material using the lowest carrier amount. The physicochemical properties of the carrier and the core-carrier ratio are two crucial factors in the selection of a suitable stabilizer. In particular, the selection of the wall materials is considered a crucial step since it determines the features and the properties of the resulting product. Natural polysaccharides are food grade, biodegradable, generally stable in food systems during processing and storage, and suitable for different industrial applications, both in pharmaceutical and nutraceutical fields [6]. Therefore, they represent a good candidate as carriers; in addition, they have a good chemical stability to high temperature and are able to entrap and retain hydrophilic and lipophilic compounds through linkage with different functional groups [7]. To date, several polysaccharides, such as guar gum or Arabic gums, starches, and pectins, have been widely applied to entrap anthocyanins [8,9]. Furthermore, some innovative polysaccharides-based delivery systems have been recently developed using xylans or heteropolysaccharide fractions from agro-food by-products [10,11].

Our previous investigation into the bioaccessibility and the solid-state stability of an anthocyanin rich extract obtained from Moradyn purple corn cobs (MCE) highlighted the instability to basic pH, temperature, and humidity [12,13], as already reported for other anthocyanin-rich extracts [14,15,16]. Therefore, the aim of the present study was to investigate the potential application of three different vegetable alcohol-insoluble polysaccharide fractions isolated from typical Lombard crops and their by-products, such as common bean seeds, exhausted corn cob, and camelina cake, as carriers. The exhausted corn cob and the camelina cake were by-products obtained from Moradyn cob processing and camelina oil production, respectively. Conversely, common bean flour was obtained from Lombard common bean seeds and was selected taking into account that other natural stabilizer agents belonging to the *Fabaceae* family have already been used and are present on the market (guar gum, Arabic gum, and soy seed derivatives).

Nine different ingredients based on different ratios of MCE and polysaccharide fraction were investigated for their encapsulation efficiency. After selecting the most promising polysaccharide fraction, CD, NIR, and FT-IR spectroscopy analyses were performed to gain a better insight into its composition and its interaction with MCE in the resulting ingredients. 

The stabilizing capacity of the selected delivery system was further assessed by applying an innovative stress-testing protocol, based on an isoconversion approach, as recently set up [13]. 

## 2. Materials and Methods

### 2.1. Vegetable Materials and Chemicals

Moradyn corn cobs (*Zea mays* L. cv Moradyn), camelina cake (*Camelina sativa* L. Crantz), common bean seeds (*Phaseolus vulgaris* L.), and Arabic gum were kindly provided by FlaNat Research s.r.l. (Milano, Italy).

Ethanol (96% *v*/*v*), magnesium chloride, sodium chloride, calcium hydroxide, HPLC-grade formic acid, methanol, and acetonitrile were purchased from Carlo Erba (Milan, Italy). Water was obtained from a Millipore Direct-QTM system (Merck-Millipore, Milan, Italy). Cyanidin-3-*O*-glucoside (C3G) was purchased from Extrashynthese (Genay, France).

### 2.2. Anthocyanin Moradyn Corn Cob Extract (MCE) Preparation

Chopped Moradyn corn cobs were extracted using an aqueous ethanol solution (50/50, *v*/*v*) at 50 °C for 3 h as reported by Ferron et al. (2020) [12]. The resulting mixture was filtered and, after the removal of the organic solvent under reduced pressure at 40 °C (Buchi R-II, Büchi Labortechnik AG, Flawil, Switzerland), the solid fraction was dried in a vacuum-drying oven at 40 °C for 48 h (Goldbrunn 1400, Expondo GmbH Köpenicker, Berlin, Germany). The dried material was stored at 4 °C and then used in all the experiments.

### 2.3. Extraction of Alcohol-Insoluble Polysaccharide Fractions

Moradyn purple cob polysaccharide fraction was isolated from exhausted raw material (EMCP). Briefly, exhausted cobs achieved through the hydroalcoholic extraction of MCE were obtained following an alkaline protocol by adding a 0.2% calcium hydroxide solution [16]. Camelina cake polysaccharide fraction (CCP) was extracted from the cake freshly obtained from the cold-press oil production process, using water as solvent. The same solvent was also used to extract the common bean seed polysaccharide fraction (CBP) from dried common bean-seed flour [16,17]. 

Different temperature, extraction time, and solvent to raw material ratio conditions were investigated for all the tested polysaccharide sources, and the extracts were filtered using a muslin cloth filter before the alcohol-insoluble polysaccharide fraction separation by adding a 96% ethanol solution (1:2.5, *v*/*v*) at 4 °C overnight. The polysaccharide fractions were separated by centrifugation at 5000 rpm for 10 min (Neya 8 ZFKN-39276, Remi Eletrotechnik LTD, Mumbai, India) and the obtained pellets were freeze dried [9,18].

The total polysaccharide yield was gravimetrically determined and expressed as the percentage of dry polysaccharide fraction (g) extracted from dry raw material (g).

#### Optimization of Polysaccharide Fraction Extraction by Response Surface Methodology (RSM)

The effect of each extraction parameter was investigated using the RSM model. The ranges of the independent variables (time, temperature, Ca^2+^ loading percentage, and solvent to raw material ratio) were selected considering preliminary experiments and literature data [9,17,19,20]. A central composite design (CCD), including the analysis of the center point in triplicate, was adopted to optimize the extraction conditions for each raw material. 

To optimize the EMCP extraction process, only extraction time in the range 5–12 min and Ca^2+^ loading on dry raw material in the range 1.2–4% (*w*/*w*) were considered, while temperature was fixed at 120 °C [21]. Considering the low water solubility of calcium hydroxide, exhausted corn cobs were always soaked in an adjusted 0.2% calcium hydroxide solution volume to obtain the required calcium hydroxide content.

For CCP, the solvent to raw material ratio was fixed at 10 (the lowest suitable ratio for the extraction), while the effects of extraction time (ranging from 5 to 15 min) and temperature (ranging from 90 to 115 °C) were investigated during the optimization step. Finally, for CBP, the optimization was performed in the extraction time range 30–120 min, temperature range 30–70 °C, and 5–10 solvent to raw material ratio.

### 2.4. MCE Encapsulation

The polysaccharide fraction (EMCP, CCP, and CBP) obtained from each raw material was suspended in water at 10% final concentration (*w/v*) and after a complete rehydration at 4 °C overnight mixed with MCE 10% (*w/v*) aqueous solutions in the ratio 70/30, 50/50, and 25/75 (*v*/*v*). Nine different ingredients were obtained; namely, MCE-EMCP (70/30), MCE-EMCP (50/50), MCE-EMCP (25/75), MCE-CCP (70/30), MCE-CCP (50/50), MCE-CCP (25/75), MCE-CBP (70/30), MCE-CBP (50/50), and MCE-CBP (25/75). All the ingredients were dried in a drying oven under vacuum (42 °C, 8 mbar) for 48 h (Goldbrunn 1400, Expondo GmbH Köpenicker, Berlin, Germany).

### 2.5. Determination of Total Anthocyanins Content in the Ingredients by RP-HPLC-WVD Analysis

To quantify the total encapsulated anthocyanin content in each ingredient, it was necessary to destroy the particles/wall material formed by the carrier with solvent. Thus, polyphenols were extracted from the ingredients following a procedure described by Norwaek et al. (2019) [22] with slight modifications: 5 mg of each dried ingredient was dissolved in 1 mL deionized water, mixed, and then sonicated for 20 min; a methanol/acetonitrile/formic acid (60:35:5, *v*/*v*/*v*) mixture was added to reach a 10 mL final volume. Samples were concentrated up to 0.25 mL under reduced pressure at 40 °C (Buchi R-II, Büchi Labortechnik AG, Flawil, Switzerland) and then diluted to a 5 mL final volume with the HPLC mobile phase (0.1% formic acid aqueous solution and 0.01% formic acid in acetonitrile, 80:20 (*v*/*v*)) before the analysis.

HPLC analyses were carried out on an Agilent Technologies 1260 Infinity II technology series system (Agilent Technology, Santa Clara, CA, USA), including a quaternary gradient pump, a vial sampler, a degasser, a thermostat column oven set at 25.0 ± 0.5 °C, and a variable wavelength detector (VWD). The HPLC-VWD system was controlled using a personal computer equipped with Agilent OpenLab CDS ChemStation software Windows 10. The chromatographic separation and anthocyanins quantification were carried out using a validated method, as previously reported by Ferron et al. (2021) [14]. Chromatograms were recorded at 520 nm.

### 2.6. Determination of Encapsulation Efficiency

The encapsulation efficiency was determined following the procedure applied by Ariyaratha and Karunaratne (2015) [23] with slight modifications: each ingredient was suspended in methanol/water solution (30:70, *v*/*v*) at the final concentration of 5 mg/mL and then centrifuged at 25 °C, 4000 rpm for 10 min (Neya 8 ZFKN-39276, Remi Eletrotechnik LTD, Mumbai, India). Supernatants were collected and the surface anthocyanin content was quantified by HPLC-VWD analysis.

The encapsulation efficiency was calculated as follows:

Encapsulation efficiency % = ([A_tot_ − A_sur_]/A_tot_) × 100 (1)

where A_tot_ and A_sur_ were the total and surface anthocyanin content (mg/mL), respectively.

### 2.7. Structural Characterization of MCE, CCP, and Relative Ingredients

#### 2.7.1. Fourier Transform Infrared Spectroscopy (FT-IR)

FT-IR spectra of CCP, MCE, and the relative ingredients (MCE-CCP 70/30, 50/50, 25/75) were obtained in attenuated total reflection (ATR) using a Nicolet FT-IR iS10 spectrometer (Nicolet, Madison, WI, USA). Thirty-two acquisitions were recorded for each spectrum using the following conditions: 4 cm^−1^ spectrum resolution, 25 kHz scan speed, 1000-scan coaddition, and triangular apodization. All spectra were reported after ATR correction, smoothing, and automatic baseline correction using OriginLab^TM^ 8 software. 

#### 2.7.2. Circular Dichroism Spectroscopy (CD)

CD spectra of CCP fraction were recorded on a Jasco J-815 (Jasco Corp., Tokyo, Japan) spectropolarimeter using a 0.1 mm quartz cuvette. Spectra were collected in the 180–300 nm spectral range and averaged over three scans at room temperature. All scans were carried out at a scan speed of 50 nm/min, with a band width of 1 nm and time–response parameters set to 2 s. A reference spectrum of distilled water was recorded and subtracted from each spectrum. The estimation of the protein secondary structure was achieved using a chemometrics method (http://bestsel.elte.hu/index.php (accessed on 30 March 2022)). All spectra were reported using OriginLab^TM^ 8 software. 

#### 2.7.3. MicroNIR Spectroscopy

NIR spectra of CCP fraction were recorded on a MicroNIR device (Viavi Solutions, JDSU Corporation, Milpitas, CA, USA) operating in the spectral region 900–1700 nm. The MicroNIR instrument is an ultra-compact device consisting of a linear variable filter (LVF) as a dispersing element directly connected to a 128 pixel linear indium gallium arsenide (InGaAs) array detector and two tungsten light bulbs as a radiation source. Collection of spectra was performed with a nominal spectral resolution of 6.25 nm (the most performing condition), using a special tool designed to get the optimal focal point, thus improving the chip sensitivity. Spectralon was used as NIR standard reference with a 99% diffuse reflectance. All spectra were recorded with an integration time of 10 ms, resulting in a total measurement time of 2.5 s per sample. Chemometric analysis was performed by using MicroNIR Pro software (JDSU Corporation, Milpitas, CA, USA).

### 2.8. Solid-State Stability Assessment by Stress Testing

The solid-state stability of each ingredient was estimated by applying an innovative stress testing protocol, developed to assess MCE stability [13] and based on the isoconversion approach [24].

Following this protocol, five different temperature (T) and relative humidity (RH) stress conditions (80 °C—30% RH, 70 °C—30% RH, 70 °C—75% RH, 45 °C—75% RH, 25 °C—75% RH) were reproduced using a temperature-controlled oven (Goldbrunn 1400, Expondo GmbH Köpenicker, Berlin, Germany) in presence of saturated salt solutions. Twenty milligrams of each ingredient was weighted in a 15 mL open transparent glass vial and submitted to the above-reported stress conditions; the degradation kinetics of each ingredient were estimated, monitoring C3G degradation at five different time points (1, 3, 6, 24, 30 h for 80 °C—30% RH; 6, 24, 48, 72, 96 h for 70 °C—30% RH; 3, 6, 24, 30, 48 h for 70 °C—75% RH; 6, 24, 96, 120, 144 h for 45 °C—75% RH and 4, 8, 10, 14, 21 days for 25 °C—75% RH). At the end of each monitored period, samples were immediately dissolved in HPLC mobile phase (0.1% formic acid aqueous solution and 0.01% formic acid in acetonitrile, 80:20 (*v*/*v*)) and the total anthocyanin content analyzed following the same procedure mentioned above. The degradation percentage was calculated by comparing the anthocyanin content at each monitoring time with the content of an untreated sample (T0).

### 2.9. Statistical Analysis

Matlab statistical software version R2019B was used to build the solid-state stability model, based on moisture-corrected Arrhenius equation, and for ANOVA testing.

The experimental design and regression analysis were performed using a Chemoface v1.64, a MATLAB standalone application for chemometrics.

## 3. Results and Discussion

### 3.1. Optimization of Alcohol-Insoluble Polysaccharide Fraction Extraction by RSM

#### 3.1.1. Exhausted Moradyn Purple Cob

Corn cobs are rich in lignocellulosic components, particularly heteropolysaccharides belonging to the xylan polymer subgroup, which represents the most relevant fraction of cell wall hemicellulose [25]. Xylan extraction usually requires the breaking of covalent bonds between polysaccharides and lignin residues and a steam explosion or a pretreatment with ultrasounds or alkaline medium at temperature higher than 100 °C [26]. 

A 0.2% calcium hydroxide solution was used to permeate exhausted corn cob at different loading ratios to set up EMCP extraction. Calcium hydroxide was preferred to sodium hydroxide, as it is considered safer, and it is already widely applied in alkali cooking procedure. Its presence in the extraction mixture should ensure the interaction of calcium cations with the polysaccharide acidic groups, thus leading to their release from the cell wall and enhancing their extraction [27]. 

Hemicellulose or heteroxylans extraction from highly lignocellulosic biomass such as corn cobs could be enhanced by temperature, but at temperature higher than 120 °C, water is subjected to an ionization process, generating H_3_O^+^ ions which induce partial polysaccharide depolymerization [19]. Therefore, preliminary single-factor experiments were carried out at 120 °C to assess the effect of time (three different values were selected; namely 5, 10, and 20 min) on extraction yield in order to define the time range to be used during the optimization step. Considering the data reported in the literature, polysaccharide extraction yield was monitored at each time both in the absence and in the presence of calcium hydroxide using a 2% Ca^2+^ loading percentage on dry raw material [26]. Significant higher extraction yields (*p* < 0.05) in the range between 0.67% ± 0.03 (*w*/*w*) and 2.32% ± 0.33 (*w*/*w*) were obtained in the presence of 2% calcium hydroxide, confirming that extraction yield was strictly related to Ca^2+^ loading capacity. Conversely, time had a negative effect on the response, since it decreased from 2.32% ± 0.33 after 10 min to 0.67% ± 0.03 after 20 min (Figure S1, Supplementary Material). This was probably due to a strong depolymerization occurring under such alkali conditions for a longer time (20 min), as already reported by Mihiretu et al. (2019) [26].

**Figure 1:**
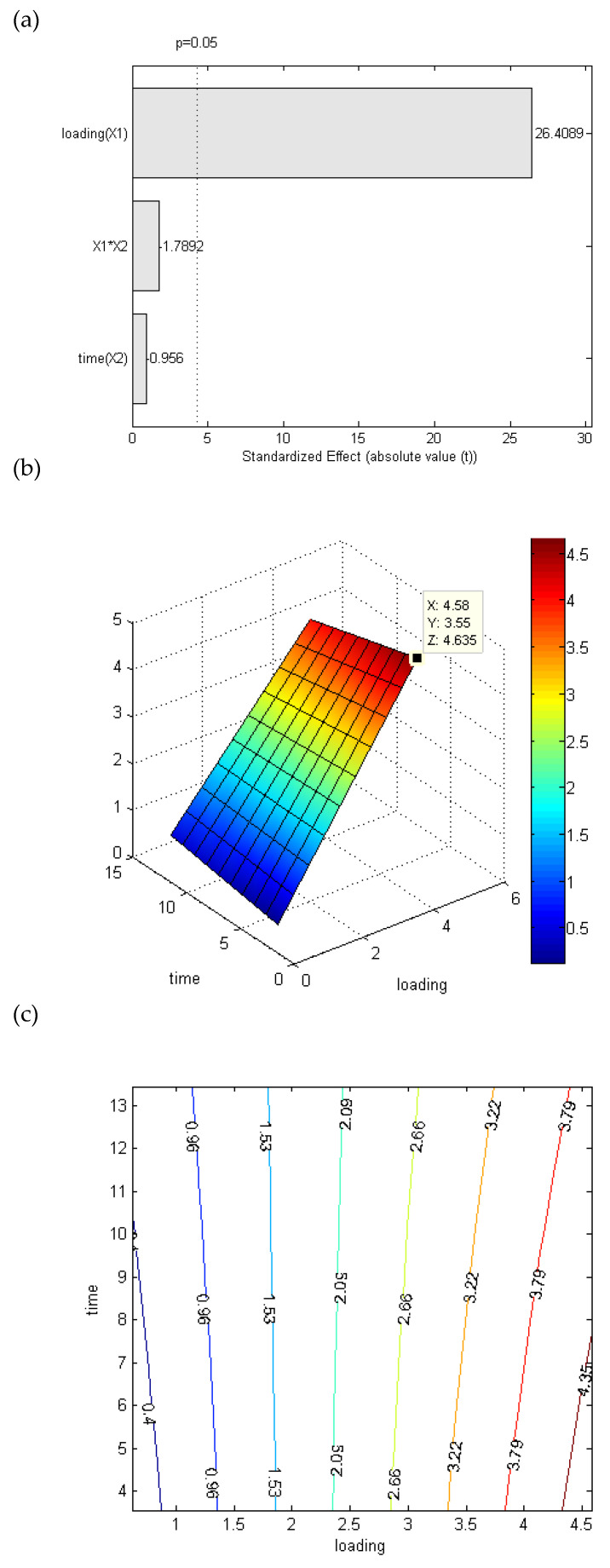
Optimization of EMCP extraction from exhausted Moradyn purple cob. (**a**) Pareto chart graphically describing the effect of each variable or their interaction on EMCP extraction yield. The vertical line indicated the significance of each effect (*p* = 0.05). (**b**) Three-dimensional response surface plot (X = Ca^2+^ loading, Y = time, and Z = yield) and (**c**) contour plot for the interactive effects of Ca^2+^ loading (X1) and extraction time (X2) in EMCP extraction system.

Based on these preliminary data, the extraction process was optimized using a two-factor central composite design considering time (5–12 min) and Ca^2+^ loading on dry raw material (1.2–4%, *w*/*w*) as independent variables, while temperature was fixed at 120 °C. The experimental conditions applied in each process and the corresponding polysaccharide extraction yields are reported in Table S1 (Supplementary Material). The effect of the two independent variables on EMCP extraction yield is summarized in the Pareto chart (Figure 1a), where each bar in the chart expresses the magnitude of the effect exerted by each parameter, or by parameters interaction, the response and their significance (*p* = 0.05, graphically indicated by the dashed line).

As reported in Figure 1a, the Ca^2+^ loading was the only significative parameter and had a strong positive effect on the yield. Nevertheless, the two independent variables were correlated to the response (yield) following an interaction model (Equation (2)), since it showed the best fit with the experimental data (determination coefficient of 0.9339).

Y = −0.7915 + 1.2382 X_1_ + 0.057 X_2_ − 0.0275 X_1_ X_2_ (2)

where Y is the yield (%) calculated from the regression model, while X_1_ and X_2_ are the coded variables of the loading and extraction time, respectively.

In order to assess the significance of the regression, the analysis of variance (ANOVA) of the response surface for the interaction model was performed, obtaining a high F value (32.982) and a low *p* value (*p* < 0.01), indicating that the model was highly statistically significant. A lack-of-fit test was further performed, confirming ANOVA analysis results and the high-quality fit of the model equation. The three-dimensional response surface (Figure 1b) and two-dimensional contour plots (Figure 1c) were plotted using Chemoface software in order to visualize the predicted model and reach the optimal extraction conditions. These were found at 3.55 min (Y axis, Figure 1b) and 4.58% Ca^2+^ loading (X axis, Figure 1b), with 4.635% (*w*/*w*) maximum predicted yield. To validate the model equation, verification experiments under these conditions were carried out and EMCP experimental extraction yield was 4.013% ± 0.084 (*w*/*w*) (mean ± RSD, *n* = 3), confirming the predicted value and therefore that the regression model was well fitted.

The obtained extraction yields were far lower than those reported in literature for corn cobs [28], probably due to the use of calcium hydroxide instead of sodium hydroxide. Ca^2+^ loading percentage higher than 4.58% could be required to improve xylans extraction yields; however, the use of such high Ca^2+^ loading percentage required an extremely high solvent to raw material ratio, making this extraction protocol unsuitable for industrial purposes.

#### 3.1.2. Camelina Cake (CCP)

Camelina is an oleaginous plant belonging to *Brassicaceae* family, differing from other members of this subgroup for its high polysaccharide or mucilage content, which is very similar to flax (*Linum usitatissimum*) [18]. The flaxseed mucilage is a “weak gel like” matrix suitable for several food and non-food applications, and its structure has been widely investigated: D-xylose, L-arabinose, D-glucose, galactose, D-galacturonic acid, and L-rhamnose were identified as the main monosaccharide constituents of this fraction, generally extracted following a water-based process under high temperature (>90 °C) [29]. Based on this similarity, preliminary experiments were performed on camelina cake to evaluate the effect of solvent to raw material ratio, which was tested in the range 3–10 *v*/*w* dry material. When ratios lower than 10 were used, camelina cake rapidly absorbed water and it was impossible to calculate any yield; thus, the solvent to raw material ratio of 10 (*v*/*w* dry material) was the lowest suitable for the extraction.

**Figure 2:**
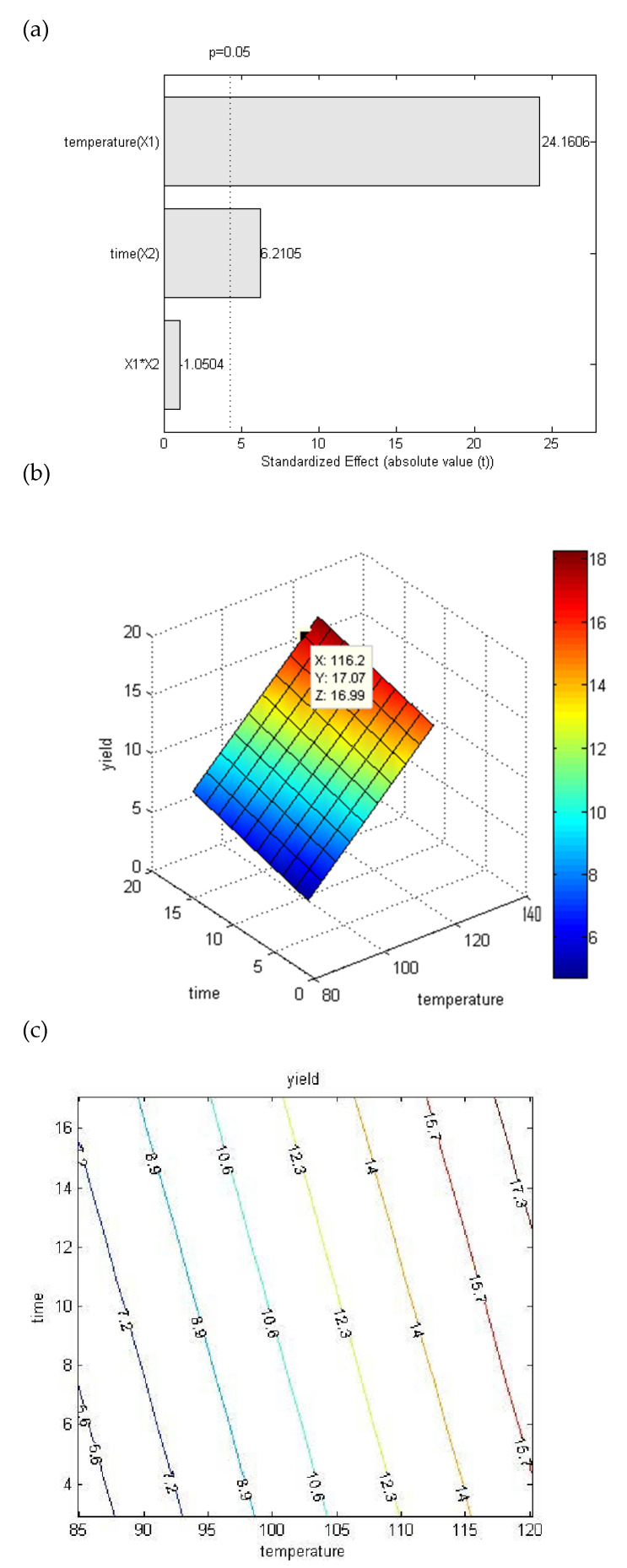
Optimization of CCP extraction from camelina cake. (**a**) Pareto chart graphically describing the effect of each variable or their interaction on CCP extraction yield. The vertical line indicated the significance of each effect (*p* = 0.05). (**b**) Three-dimensional response surface plot (X—temperature, Y—minutes, Z—yield) and (**c**) contour plot for the interactive effects of extraction temperature (X1) and time (X2) in CCP extraction system.

A two-factor central composite design was applied to optimize the extraction temperature and time, ranging between 90 and 115 °C and between 5 and 15 min, respectively. The applied experimental conditions and the relative obtained yields are listed in Table S2 (Supplementary Material). The effect of the two variables on CCP extraction yield are summarized in Figure 2a showing the Pareto chart: both temperature and time had a strong positive effect on yields, which resulted in about 15.84% (*w*/*w*) at 115 °C after 15 min of extraction. This value was higher than those generally reported for flax, which ranged between 3.3 and 5.5% when calculated on dry seeds weight [29]. However, the interaction between the two variables was not significative, and therefore they were correlated to the response (yield) following a linear model (Equation (3), determination coefficient value 0.9474):

Y = −21.5308 + 0.3028 X_1_ + 0.1946 X_2_ (3)

where Y is the yield of CCP (%) calculated from the regression model, while X_1_ and X_2_ are the coded variables of the extraction temperature and time, respectively.

This model resulted as statistically significant (*p* < 0.01 and F = 72.073) with a non-significative lack of fit, further confirming the high-quality fit of the model equation.

The optimal extraction conditions were extrapolated from the three-dimensional response surfaces and two-dimensional contour plots reported in Figure 2b,c, respectively. Therefore, the model was validated by performing three independent extraction processes at 116.2 °C (X axis, Figure 2b) and 17.1 min (Y axis, Figure 2b). The experimental mean yields were 16.50% ± 0.49 (*w*/*w*) (mean ± RSD, *n* = 3) vs. a maximum predicted yield of 16.99% (*w*/*w*), highlighting the accuracy and adequacy of the model.

#### 3.1.3. Common Bean Seeds (CBP)

In contrast, the CBP fraction extraction process was optimized using a three-factor central composite design and the considered independent variables were extraction temperature in the range 30 °C–60 °C, time in the range 30–120 min, and solvent to raw material ratio between 5 and 10, which were fixed on the basis of a procedure already reported by Yi et al. (2016) [20]. The experimental conditions and yields are reported in Table S3 (Supplementary Material).

**Figure 3:**
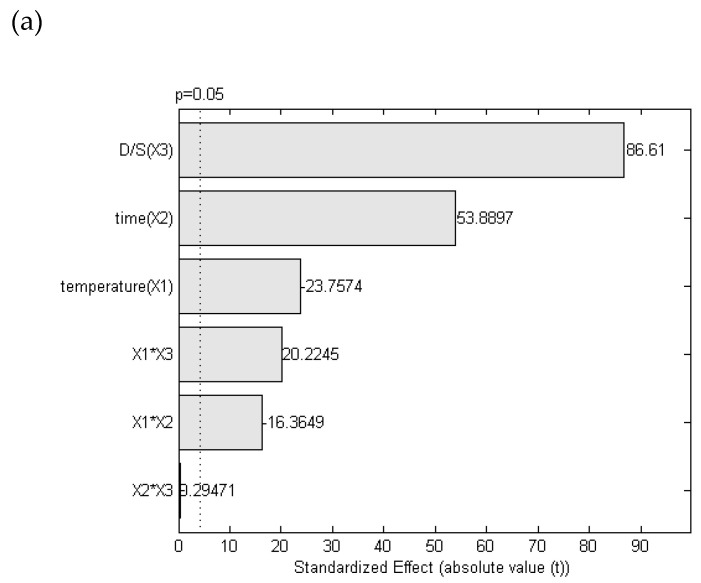

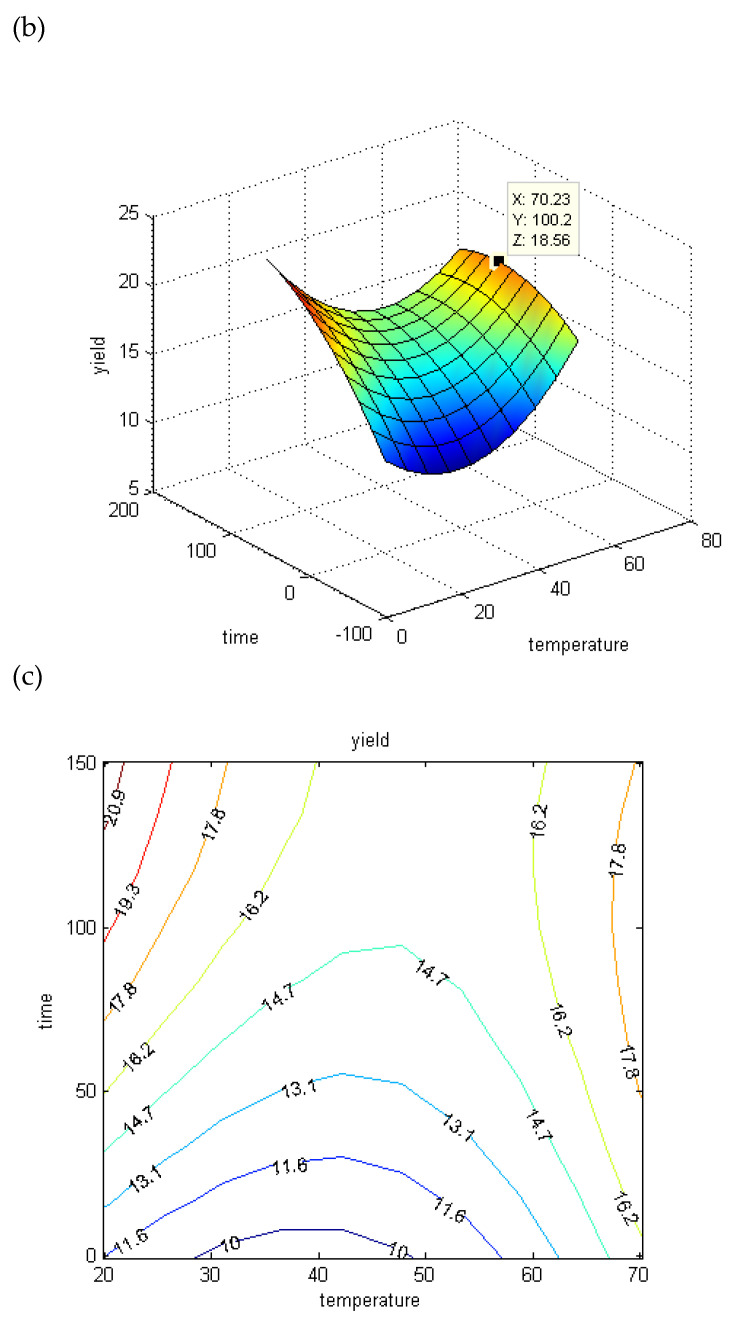
Optimization of CBP extraction. (**a**) Pareto chart graphically describing the size of the effect of each variable or their interaction on CBP extraction yield. The vertical line indicated the significance of each effect (*p* = 0.05). (**b**) Three-dimensional response surface plot (X—temperature, Y—time, X—yield) and (**c**) contour plot for the interactive effects of extraction temperature (X1), time (X2), and solvent to raw material ratio (X3), set at 10 in CBP extraction system.

As evident from the Pareto chart (Figure 3a), all the considered parameters had a significative effect on the response; in particular, solvent to raw material ratio and time had stronger positive effects, differently from temperature, negatively affecting the extraction yield. These three independent variables and their interactions were correlated to the response (yield) using a quadratic model (Equation (4)).

Y = 24.023 − 0.7446 X_1_ + 0.1245 X_2_ − 1.6346 X_3_ − 0.001 X_1_ X_2_ + 0.0234 X_1_ X_3_ + 0.000114 X_2_ X_3_ + 0.0066 X_1_^2^ − 0.00025 X_2_^2^ + 0.115 X_3_^2^ (4)

where X_1_ is Temperature, X_2_ time, and X_3_ the solvent to raw material ratio.

This model was the best fit with experimental data, even if it is not significant, as is evident from the low R^2^ value obtained (0.638) and the ANOVA analysis. This was probably due to the complexity of this raw material, which is particularly rich both in globular proteins (34% on dry weight) and pectin-like polysaccharides (60% of total pectin amount in cell walls) [17]. At high temperature (≥70 °C), strongly enhancing polysaccharide extraction, proteins could lose their structure and solubility properties and did not precipitate by adding ethanol. Conversely, at low temperature, the extracted water-soluble proteins maintained their physicochemical properties and could be partitioned into the alcohol-insoluble fraction [17]. So, considering that the extraction yields obtained at low temperature (<30 °C) after 60 min were very similar to those obtained after 50 min at 70 °C (Figure 3b,c), it could be speculated that these yields were related to high protein or polysaccharide content, respectively; therefore, the composition of alcohol-insoluble fraction extracted at low temperature could not be compared with that of the fraction obtained under high temperature, highlighting the inadequacy of the gravimetric method to quantify the alcohol-insoluble polysaccharide fraction from common bean during this experimental design.

Nevertheless, the selected extraction conditions were 70 °C for 100 min, and solvent to raw material ratio 10/1 (Figure 3b).

### 3.2. Encapsulation Efficiency

MCE encapsulation efficiency (EE%) in each ingredient obtained using 30, 50, or 75% EMCP, CCP, and CBP (*w*/*w*) was evaluated (namely, the samples were MCE-EMCP (70/30), MCE-EMCP (50/50), MCE-EMCP (25/75), MCE-CCP (70/30), MCE-CCP (50/50), MCE-CCP (25/75), MCE-CBP (70/30), MCE-CBP (50/50), MCE-CBP (25/75)) by monitoring anthocyanin content and compared with the value obtained using Arabic gum as carrier at the same concentrations reported for polysaccharide carriers (Figure S2, Supplementary Material).

As regards EMCP-based ingredients, no anthocyanins were detected, probably because calcium hydroxide residues were still present in the polysaccharide fractions after ethanolic precipitation, and therefore during blend preparation, an alkaline environment (pH 10.43 ± 1.04) was created, and anthocyanins degraded.

As regards CCP and CBP, their EE% values were very similar and always significantly higher (*p* < 0.05) than those of Arabic gum at all tested concentrations, showing EE% higher than 60% and 80% when present in the ingredients at 50 and 75% (*w*/*w*), respectively. Therefore, the core/wall ratio had a strong effect on the encapsulation efficiency, that increased by increasing this ratio, and reached the best condition when a 25/75 core/wall ratio was used, as already observed by Shu, Yu, Zhao, and Liu (2006) [30] for lycopene encapsulation.

The EE trend registered for CCP and CBP was close to that reported for other commercial polysaccharide mixtures consisting of guar gum and Arabic gum, maltodextrins, or inulin, used with a core/carrier ratio of 1/3 or higher ratios [31,32].

Moreover, results obtained for 75% CCP or CBP ingredients were similar to those reported by Mahadavi et al. (2016) [33], who compared the EE% of an anthocyanin-rich extract from barberry (*Berberis vulgaris*) using maltodextrin, Arabic gum, gelatin, and their combinations, thus obtaining 89.06–96.21 EE%.

However, to the best of our knowledge, the EE values registered for the ingredients containing 50% CCP or CBP have never been obtained using commercial carriers or their mixtures at the same core/carrier ratio, thus supporting the high efficiency of these polysaccharide fractions.

Based on these results, and on the fact that CCP can be considered a more added-value ingredient since it is obtained from a by-product, and therefore it can meet the request of the circular economy action plan approved by the European Commission [34], the following step of the research was focused on this polysaccharide and further experiments were carried out using 30, 50, and 75% CCP ingredients.

### 3.3. Physicochemical Characterization of CCP

**Figure 4:**
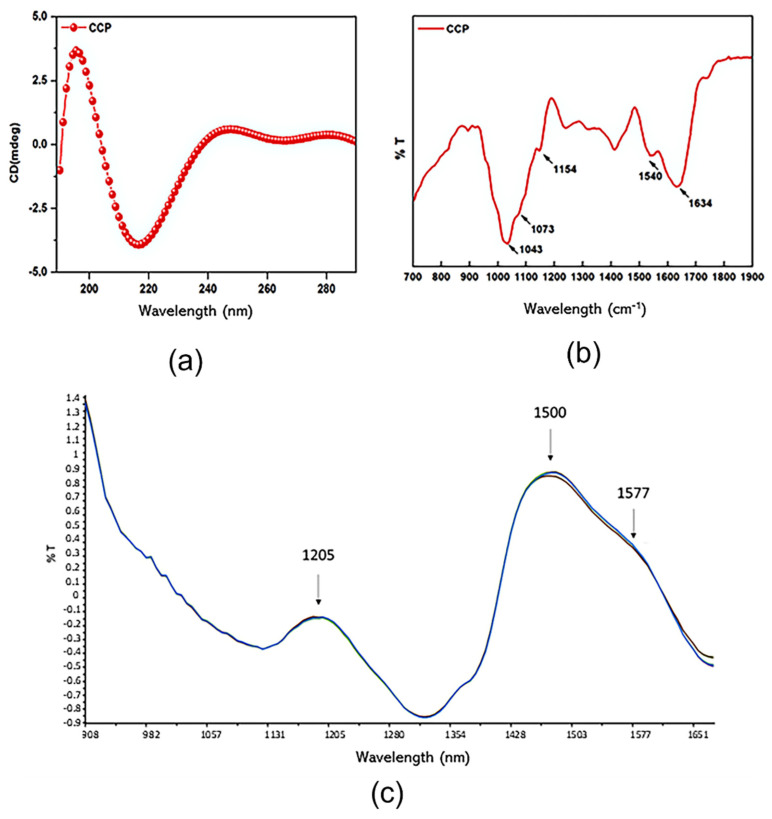
CCP structural organization. (**a**) CD spectra, (**b**) FT-IR spectrum with a characteristic β-turns/random coil conformation in the Amide I and Amide II regions at 1634 cm^−1^ and 1540 cm^−1^, respectively, and typical polysaccharides vibrations in the region 800–1200 cm^−1^. (**c**) NIR spectrum showing the fundamental absorption peaks of phenolic compounds and unsaturated fatty acids in cis conformation (1205, 1500, and 1577 nm).

Circular dichroism (CD), FT-IR spectroscopy, and microNIR spectroscopy were used to better investigate CCP composition. In detail, to explore the secondary structure conformation of the protein component in the CCP fraction, CD spectra were recorded in the far UV region of 180–300 nm (Figure 4a). A positive and negative Cotton effect was observed at 198 nm and 215 nm, characteristic of β-turns and random coil signals, respectively. To obtain further information on the secondary structure of such proteins, a chemometric approach previously reported by Raussen, Ruysschaert, and Goormaghtigh (2003) [35] was applied. This simple and rapid method allowed us to extrapolate good estimates of protein secondary structure content from CD spectra without any previous knowledge of the sample concentration. The method involved two steps: a single-wavelength normalization procedure and the application for each secondary structure of a quadratic model based on one or two wavelength intensities. The results indicated 71% random coil conformation, 14% β-turns structures, 6% antiparallel β-sheet structures, and 9% α-helical structures.

FT-IR spectra (Figure 4b) registered for CPP showed one broad Amide I peak at 1634 cm^−1^ and a lower stretching peak at 1540 cm^−1^ in the Amide II region, characteristic of the β-turns/random coil conformation, in agreement with the CD spectra results. Instead, in the region of the polysaccharide stretching vibrations (800–1200 cm^−1^), an intensive peak centered at 1043 cm^−1^ ascribed to the mannose, arabinose, and rhamnose constituents was observed [36], in addition to two less intense peaks at 1073 cm^−1^ and 1154 cm^−1^, indicating galactose unit and xyloglucan sub-unit presence, respectively [36].

Considering that camelina seeds composition is mainly characterized by polysaccharide and proteins [18], the data obtained from FT-IR analysis pointed to the fact that the isolation of CCP by alcohol precipitation involved also alcohol-insoluble proteins, which still maintained their solubility properties although the extraction temperature was fixed at 116.2 °C. Overall, these data suggested a gum-like composition close to that reported for Arabic gum, which is mainly composed of highly branched heteropolysaccharides coexisting with a small amount of protein covalently linked to the carbohydrate chain [9].

Regarding microNIR spectroscopy, the Standard Normal Variate (SNV) method was used to correct CPP NIR spectra in the spectral range of 900–1700 nm. CCP exhibited three fundamental absorption peaks at 1205, 1500, and 1577 nm (Figure 4c). The first peak centered at 1205 nm was related to the first harmonic of the –CH groups stretching vibrations of phenolic compounds [37]. The second peak at 1500 nm was referred to the C=C and C–H stretch of the unsaturated fatty acids in *cis* conformation. The less intense peak at 1577 nm was ascribed to the –OH stretching, also visible in the FT-IR analyses at 3275 cm^−1^ (Figure S3, Supplementary Material). Therefore, the presence of fatty acids and phenolic compounds in CCP could be hypnotized consistently with camelina seeds composition [18].

### 3.4. Spectroscopy Characterization of MCE-CCP Ingredients

**Figure 5:**
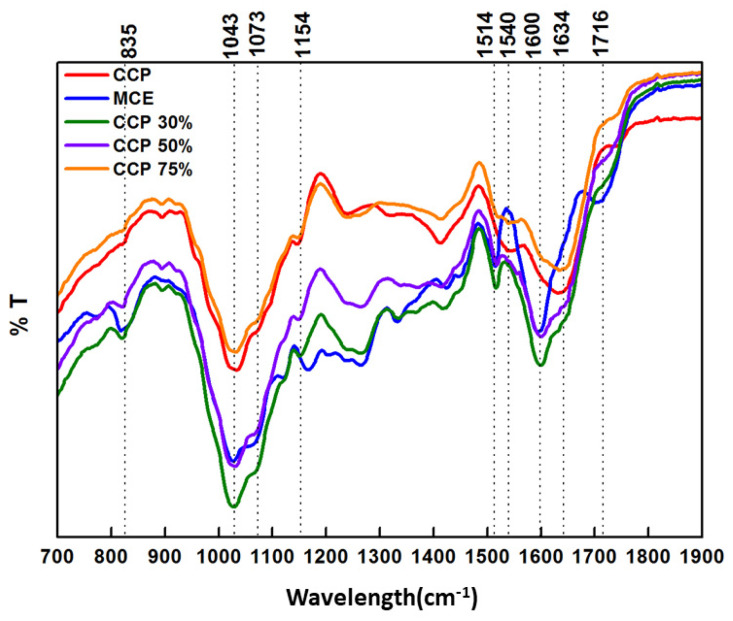
FT-IR spectra. CCP spectrum was red line, MCE blue line, MCE-CCP 30/70 green line, MCE-CCP 50/50 purple line, and MCE-CCP 75/25 orange line.

To investigate the global arrangement of MCE and CCP when used at different ratios in the ingredients, FT-IR studies were performed (Figure 5).

MCE FT-IR spectrum (blue line) showed antiparallel β-sheet features with a sharp Amide I band at 1600 cm^−1^ and a shoulder at 1716 cm^−1^. The β-sheet aggregation was also confirmed by the presence of an Amide II band at 1514 cm^−1^ directly related to CN stretching and NH bending, as previously reported [38]. Furthermore, we investigated the ratio of peak intensities at 1716 cm^−1^ and 1600 cm^−1^, named β-sheet organizational index by Sarroukh, Goormaghtigh, Ruysschaert, and Raussens (2013) [39], that is directly correlated to the ratio of antiparallel/parallel β-sheet structures, and in our case amounts to 76.33%. However, the shoulder at 1716 cm^−1^ was a specific feature of MCE and it has been also related to the C=C scissoring in phenolic groups [40], which was consistent with the previous qualitative profile of MCE obtained from RP-HPLC-DAD-ESI-MS^n^ analysis [12].

It was interesting to note that in MCE-CCP 70/30 (green line) and 50/50 (purple line) ingredients, the absorption peaks due to the Amide I and Amide II regions were similar to those observed for MCE. However, in the ingredients the β-sheet organizational index values shift to 68.58% and 69.53% for MCE-CCP 70/30 and 50/50, respectively, indicating a slight loss of stable β-sheet antiparallel structures. In the polysaccharide stretching vibrations region, the ingredients displayed all peaks observed for CCP and MCE separately (i.e., 835, 1043, 1073, and 1154 cm^−1^). Conversely, for the MCE-CCP 25/75 ingredient (orange line), one broad peak at 1634 cm^−1^ (Amide I region) and a less intense stretching peak at 1540 cm^−1^ (Amide II region), attributable to the β-turns/random coil conformation, were displayed. These data were closely correlated to those of CCP. Likewise, the same structural patterns in the polysaccharide region (800–1200 cm^−1^) were observed.

The obtained results indicated that the presence of MCE in the CCP-based ingredient did not impair its overall structure, but it led to the formation of more stable β-sheets secondary structures and α-glycosidic linkage.

### 3.5. Solid-State Stability Assessment

Considering the encapsulation efficiency of CCP-based ingredients, the solid-state stability studies were carried out only on MCE-CCP 50/50 and 25/75 ingredients.

The stabilizing properties of 50% and 75% CCP were evaluated using an innovative stress-testing protocol based on the isoconversion approach introduced by Waterman (2011) and already applied for MCE [13]. Following this approach, the intrinsic stability of an active ingredient was determined considering only its isoconversion time, which is the period needed by the active monitored molecule or marker to reach its prefixed specification limit of degradation when exposed to a specific storage condition (different T and RH values).

C3G was selected as the most appropriate marker to be monitored, since its degradation rate was always faster than other flavanols characterizing MCE [13], and therefore it could be considered representative of MCE overall degradation status.

Following the isoconversion approach, C3G degradation reactions in each ingredient were monitored under five different T and RH conditions (80 °C—30% RH, 70 °C—30% RH, 70 °C—75% RH, 45 °C—75% RH, 25 °C—75% RH) (Supplementary Figure S2), and for each experimental condition the degradation rate value (k, degradation %/day) was extrapolated from the slope of the straight line passing from the origin to the point corresponding to C3G specification limit of degradation (20%) (Table 1) [13]. These extrapolated values were used to build a multilinear regression model (Figure 4 and Figure 5) and extrapolate the moisture-corrected Arrhenius equation describing C3G behavior in each ingredient.

C3G degradation in MCE-CCP 50/50 ingredient was described by Equation (5), which was characterized by an adjusted-R squared value of 0.99 and a coefficient of variation of the root mean square error (CV(RMSE)) of 0.03%; Equation (6) was related to C3G behavior in MCE-CCP 25/75 ingredient, with an adjusted-R squared value of 0.87 and CV(RMSE) of 3%. Therefore, based on these values, both these models were accurate; moreover, ANOVA analysis (*p* = 0.05) indicated *p*-values of 0.006 and 0.039 for MCE-CCP 50/50 and MCE-CCP 25/75, respectively.

Lnk = 24.209 − [7626.6 × (1/T(K))] + (0.033 × RH %)(5)

Lnk = 29.915 − [10538 × (1/T(K))] + (0.08 × RH %)(6)

The mathematical models obtained for each ingredient were applied to calculate C3G isoconversion time at 25 °C—30% RH (which mimic ingredient storage condition). The results indicated that CCP strongly improved MCE storage stability from 29.5 ± 3.5 days (for MCE) to 113.5 ± 3.1 days and 409.23 ± 36.6 days when encapsulated with 50 or 75% CCP, respectively.

These results generally agreed with data reported in the literature [15,41,42]; however, it was extremely difficult to compare results obtained by applying this protocol with those reported in literature since these last values were extrapolated following a classical, non-standardized approach. Nevertheless, it was already reported that a polysaccharide-based carrier could strongly improve polyphenols stability under high temperature [31], as occurred for CCP, especially at low RH value. Moreover, under such conditions, the C3G behavior was different in the two ingredients, since degradation rates were slower, increasing CCP concentration. Conversely, when the ingredients were stored at 75% RH, the registered degradation rates were similar (Table 1, Figure S6, Supplementary Material) and in the same order of magnitude previously registered for MCE [13]. These data supported the strong protective effect from thermal degradation during storage exerted by such kinds of polysaccharides. It is notable that under high humidity value (75%) the molecular mobility and diffusivity increased, while polysaccharide glass transition temperature decreased, thus losing stability [42].

## 4. Conclusions

Today, different carriers have been employed to encapsulate anthocyanin-rich extracts and preserve their antioxidant activities, among which vegetable polysaccharides such as Arabic gum, maltodextrins, inulin, or other purified native gums are reported to be the most compatible with hydrophilic compounds such as anthocyanins. Moreover, several research groups used protein and polysaccharide mixtures to encapsulate anthocyanins in order to exploit the complementary chemical–physical features of all the components and enhance their encapsulation efficiency and stability.

Thus, in this work, three different vegetable polysaccharide fractions were isolated and tested at different concentrations as carriers for MCE, considering not only their encapsulation efficiency, but also the sustainability of the extraction process required for their production.

CCP was selected, since it was isolated from a by-product, and it showed the highest EE% value and storage stability at the concentration of 75% (*w*/*w*), confirming the trend already registered for other polysaccharide-based wall materials. Moreover, this fraction at the concentration of 50% and 75% (*w*/*w*) strongly improved MCE solid stability under high-temperature conditions.

Preliminary FT-IR analysis on CCP suggested that this fraction could be characterized by a gum-like composition close to that reported for Arabic gum, an exceptional film-forming and encapsulating agent. Based on this, it could be concluded that CCP encapsulation efficiency and stabilizing properties (already present at 50% (*w*/*w*) in the ingredient) are better than Arabic gum, probably due to the coexistence of polysaccharides and proteins in the fraction, which creates an amorphous environment where the flavylium cation of anthocyanins becomes less vulnerable to oxygen, light, and heat, leading to an increase in the stability of the anthocyanins.

Up to now, CCP has been only partially characterized by spectroscopic techniques; therefore, the research will continue in order to fully characterize this fraction and better investigate its stabilizing properties by monitoring MCE antioxidant capacity under storage conditions.

## Figures and Tables

**Table 1:** C3G experimental k values extrapolated from the degradation kinetic curves registered for MCE-CCP 50/50 and MCE-CCP 25/75 under five different storage conditions.

Storage Condition		K Value (Degradation %/Day)	
Temperature (°C)	RH %	50% CCP	75% CCP
45	75	18.65	16.35
80	30	31.96	22.52
70	75	238.1	168.54
70	30	13.4	2.69
25	75	3.11	2.07

## Data Availability

Data is contained within the article and supplementary material.

## Supplementary Materials

The following supporting information can be downloaded at: https://www.mdpi.com/article/10.3390/antiox11050916/s1, Figure S1: EMCP extraction yields. They were obtained using 10:1 solvent (water or 2% Ca(OH)_2_ solution) to raw material ratio, at 120 °C, after 5, 10, and 20 min. Different capital letters indicate significative differences (*p* = 0.05); Figure S2: Encapsulation efficiency (EE%) registered for MCE-CCP and MCE-CBP ingredients obtained using 30, 50, and 75% (*w*/*w*) of polysaccharide carrier. Arabic gum (30, 50, and 75% (*w*/*w*)) was used as reference carrier. Different capital letters indicated significant differences between ingredients with the same core/carrier ratio (*p* = 0.05); Figure S3: –OH stretching of CCP centered at 3275 cm^−1^; Figure S4: Multilinear regression model for MCE-CCP (50/50) ingredient. (a,b) Different perspectives of a three-dimensional surface plot of the model generated by the Matlab curve-fitting tool for MCE-CCP (50/50) ingredient. (c) The residual plot generated using the same tool (*p* = 0.05); Figure S5: Multilinear ASAP model for MCE-CCP (25/75) ingredient. (a,b) Different perspectives of a three-dimensional surface plot of the model generated by the Matlab curve-fitting tool for MCECCP (25/75) ingredient. (c) The residual plot generated using the same tool (*p* = 0.05); Figure S6: Kinetic graphs of C3G degradation in MCE-CCP (50/50) (blue) and MCE-CCP (25/75) (orange) ingredients when exposed to (a) 25 °C—75% RH, (b) 45 °C—75% RH, (c) 70 °C—75% RH, (d) 70 °C—30% RH, and (e) 80 °C—30% RH. Degradation rates were extrapolated from the slope of the straight line (red line) going from the origin to the point corresponding to the specification limit of degradation; Table S1: Coded levels and real values for EMCP extraction process and yield results. Runs were reported following their standard order; Table S2: Coded levels and real values for CCP extraction process and yield results. Runs were reported following their standard order; Table S3: Coded levels and real values for CBP extraction process and yields results. Runs were reported following their standard order.
