# Supplementary material for: Selection and Optimization of an Innovative Polysaccharide-Based Carrier to Improve Anthocyanins Stability in Purple Corn Cob Extracts
Source: Antioxidants (Basel). 2022 May 6;11(5):916. doi: 10.3390/antiox11050916 (PMC9138105; doi:10.3390/antiox11050916)
Supplement: Supplementary file 1 [file antioxidants-11-00916-s001.zip › antioxidants-1674443-supplementary.pdf]

**Supplementary Table S1.** Coded levels and real values for EMCP extraction process and yield results. Runs were reported following their standard order.

| Run | Coded level (real value) | | Extraction yield (%) |
|---|---|---|---|
| | $X_1$ ($Ca^{2+}$ loading %) | $X_2$ (Time) | |
| 1 | -1 (1.2) | -1 (5.0) | 0.82 |
| 2 | -1 (1.2) | +1(12.0) | 0.83 |
| 3 | +1 (4) | -1 (5.0) | 3.81 |
| 4 | +1 (4) | +1 (12.0) | 3.28 |
| 5 | -1.414 (0.6) | 0 (9.0) | 0.68 |
| 6 | +1.414 (4.6) | 0 (9.0) | 4.80 |
| 7 | 0 (2.6) | -1.414 (4.0) | 1.78 |
| 8 | 0 (2.6) | +1.414 (14.0) | 1.86 |
| 9 | 0 (2.6) | 0 9.0 | 2.32 |
| 10 | 0 (2.6) | 0 9.0 | 2.57 |
| 11 | 0 (2.6) | 0 9.0 | 2.60 |

**Supplementary Table S2.** Coded levels and real values for CCP extraction process and yield results. Runs were reported following their standard order.

| Run | Coded level (real value) | | Extraction yield (%) |
|---|---|---|---|
| | $X_1$ (Temperature °C) | $X_2$: (Time) | |
| 1 | -1 (90) | -1 (5) | 9.16 |
| 2 | -1 (90) | +1 (15) | 7.27 |
| 3 | +1 (115) | -1 (5) | 15.30 |
| 4 | +1 (115) | +1 (15) | 15.85 |
| 5 | -1.414 (84.822) | 0 (10) | 5.29 |
| 6 | +1.414 (120.177) | 0 (10) | 16.19 |
| 7 | 0 (102.5) | + 1.414 (2.982) | 8.73 |
| 8 | 0 (102.5) | -1.414 (17.071) | 12.89 |
| 9 | 0 (102.5) | 0 (10) | 12.63 |
| 10 | 0 (102.5) | 0 (10) | 12.84 |
| 11 | 0 (102.5) | 0 (10) | 12.85 |

**Supplementary Table S3.** Coded levels and real values for CBP extraction process and yields results. Runs were reported following their standard order.

| Run | Coded levels (real values) | | | Extraction yields (%) |
|---|---|---|---|---|
| | $X_1$ (Temperature °C) | $X_2$ (time) | $X_3$ (solvent/raw material L/g) | |
| 1 | -1 (30) | -1 (30) | -1 (5) | 13.53 |
| 2 | -1 (30) | -1 (30) | +1 (10) | 14.21 |
| 3 | -1 (30) | +1 (120) | -1 (5) | 13.49 |
| 4 | -1 (30) | +1 (120) | + 1 (10) | 18.51 |
| 5 | +1 (60) | -1 (30) | -1 (5) | 9.02 |
| 6 | +1 (60) | -1 (30) | +1 (10) | 14.43 |
| 7 | +1 (60) | +1 (120) | -1 (5) | 10.43 |
| 8 | +1 (60) | +1 (120) | +1 (10) | 17.67 |
| 9 | -1.682 (19.7) | 0 (75) | 0 (7.5) | 12.86 |
| 10 | +1.682 (70.226) | 0 (75) | 0 (7.5) | 13.46 |
| 11 | 0 (45) | -1.682 (0) | 0 (7.5) | 0 |
| 12 | 0 (45) | +1.682 (150) | 0 (7.5) | 12.82 |
| 13 | 0 (45) | 0 (75) | -1.682 (3.3) | 3.71 |
| 14 | 0 (45) | 0 (75) | +1.682 (11.7) | 16.15 |
| 15 | 0 (45) | 0 (75) | 0 (7.5) | 10.85 |
| 16 | 0 (45) | 0 (75) | 0 (7.5) | 10.40 |
| 17 | 0 (45) | 0 (75) | 0 (7.5) | 10.68 |

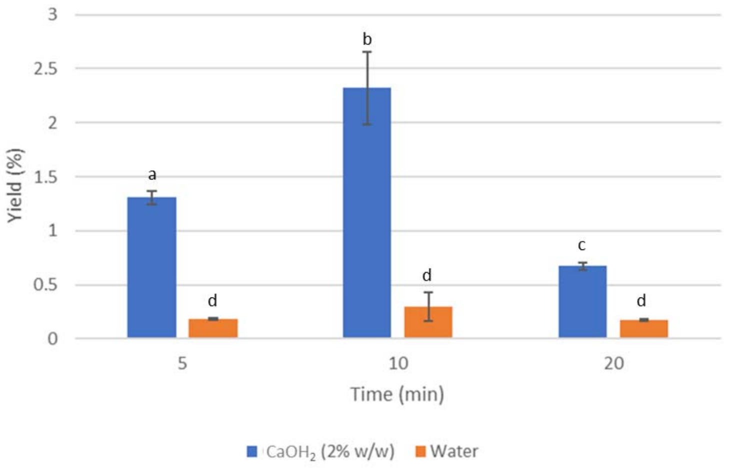


**Figure S1.** EMCP extraction yields. They were obtained using 10:1 solvent (water or 2% $Ca(OH)_2$ solution) to raw material ratio, at 120 °C, after 5, 10, and 20 min. Different capital letters indicate significative differences ($p$ = 0.05).

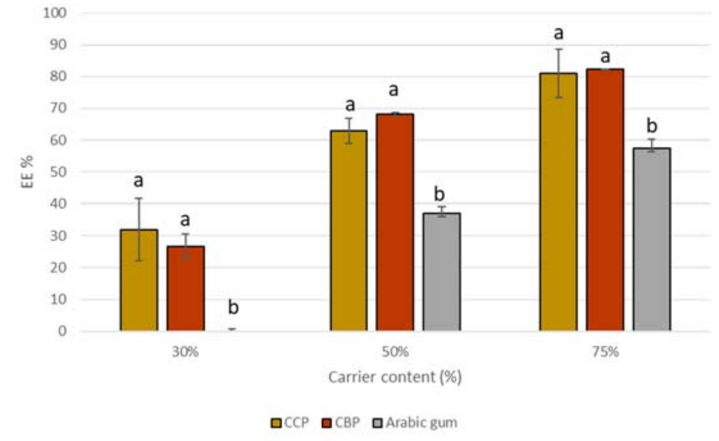


**Figure S2**. Encapsulation efficiency (EE%) registered for MCE-CCP and MCE-CBP ingredients obtained using 30, 50, and 75% (w/w) of polysaccharide carrier. Arabic gum (30, 50, and 75% (w/w)) was used as reference carrier. Different capital letters indicated significant differences between ingredients with the same core/carrier ratio ($p$= 0.05).

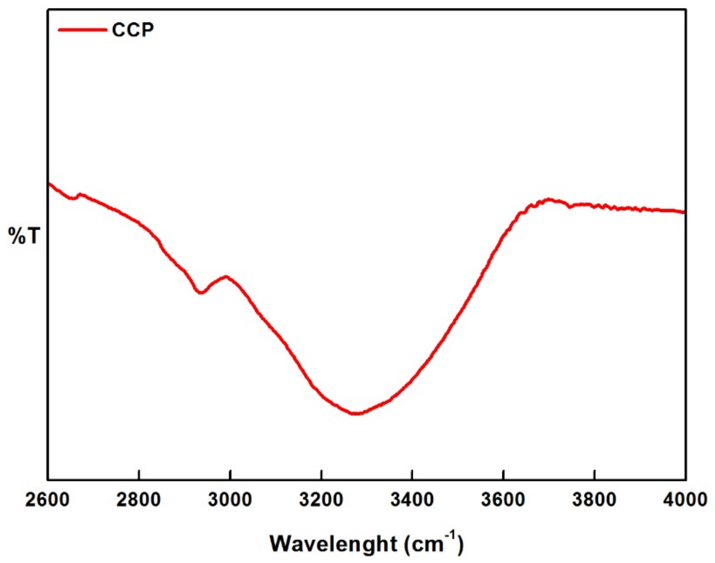


**Figure S3**. –OH stretching of CCP centered at 3,275 $cm^{-1}$.

a)

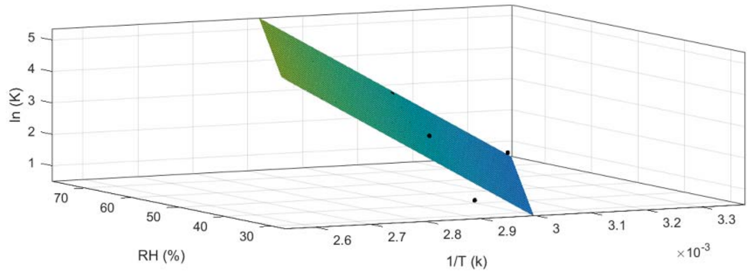


b)

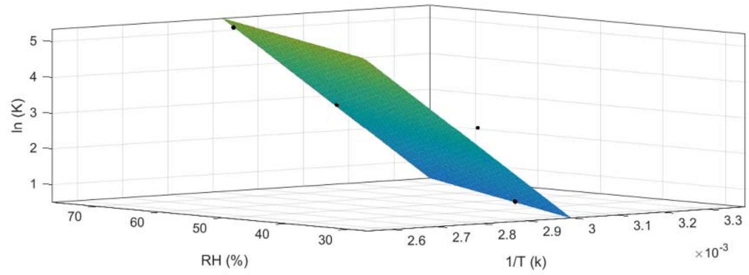


c)

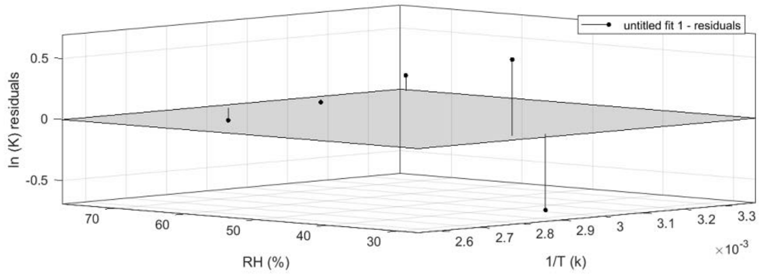


**Figure S4.** Multilinear regression model for MCE-CCP (50/50) ingredient. a) and b) Different perspectives of a three-dimensional surface plot of the model generated by the Matlab curve-fitting tool for MCE-CCP (50/50) ingredient. c) The residual plot generated using the same tool ($p$ = 0.05).

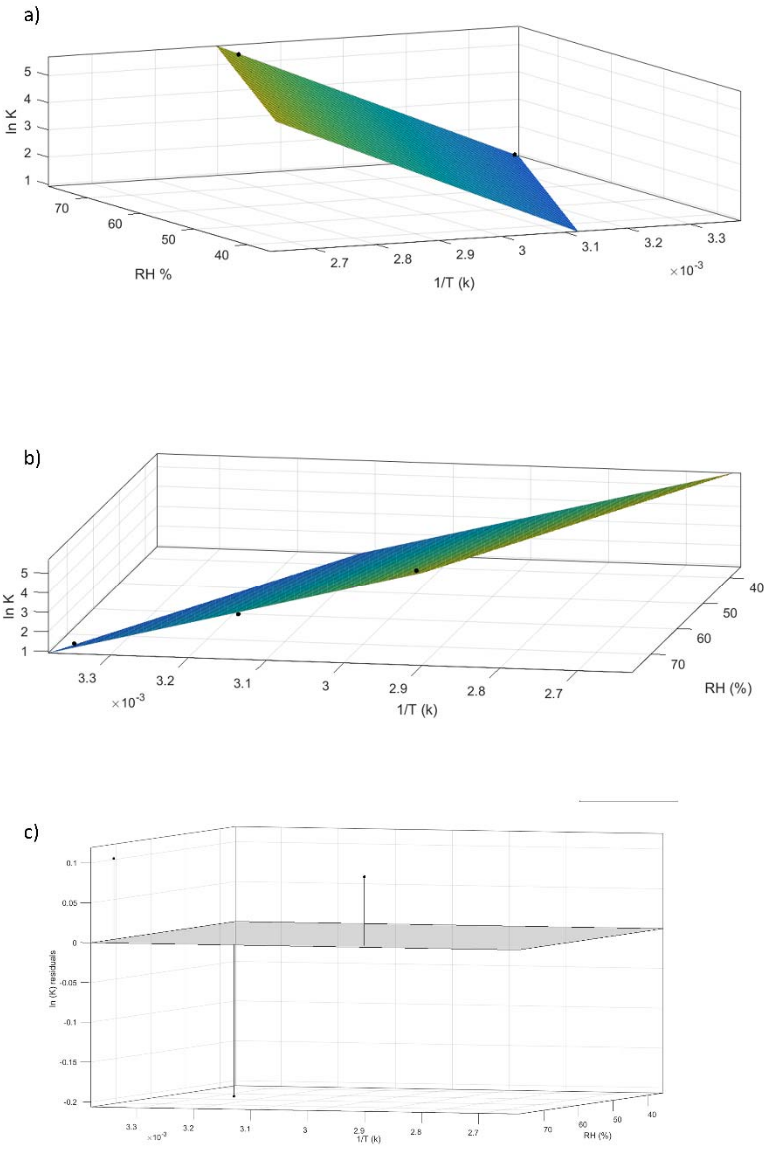


**Figure S5.** Multilinear ASAP model for MCE-CCP (25/75) ingredient. a) and b) Different perspectives of a three-dimensional surface plot of the model generated by the Matlab curve-fitting tool for MCE-CCP (25/75) ingredient. c) The residual plot generated using the same tool (*p*= 0.05).

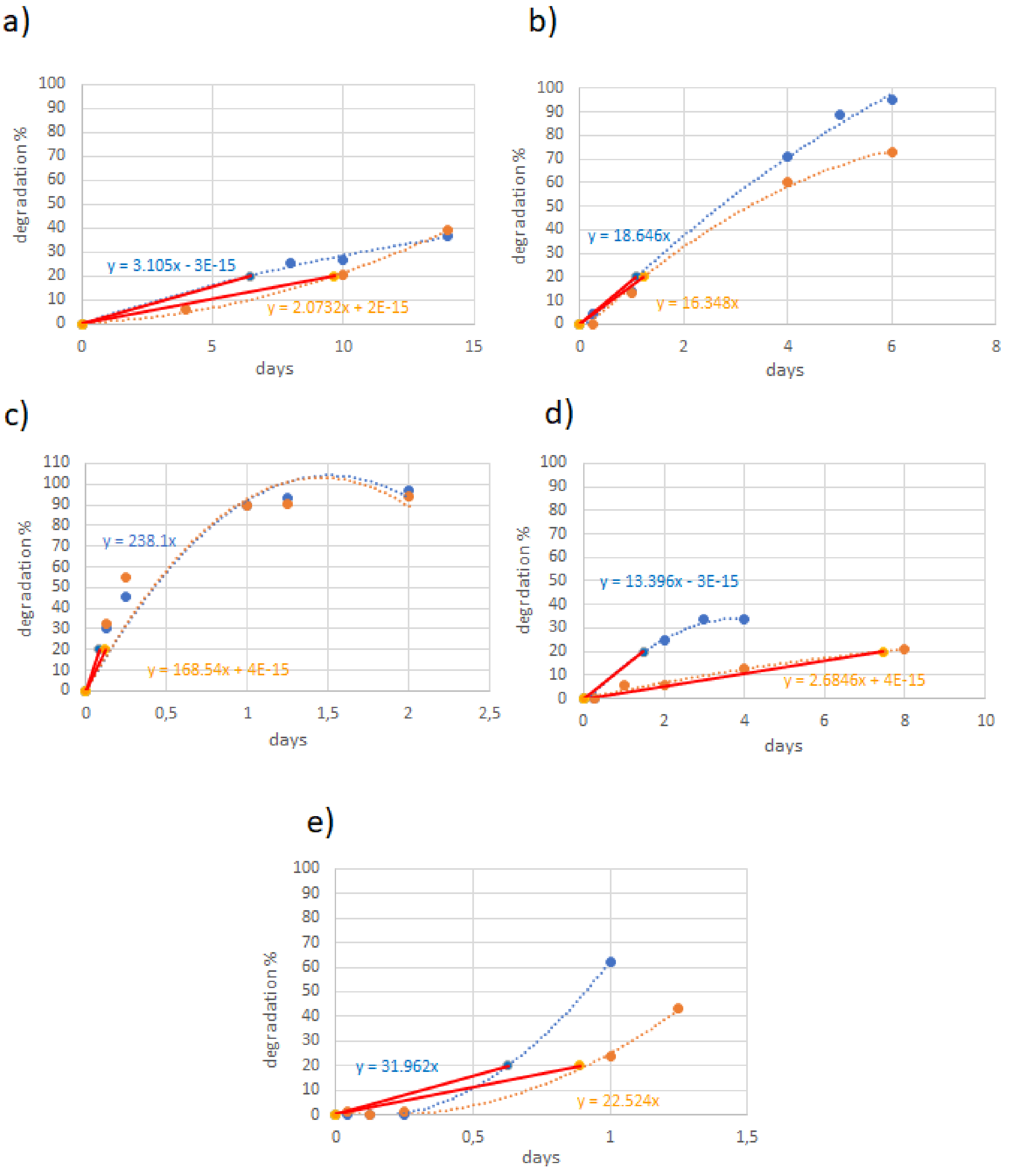


**Figure S6**. Kinetic graphs of C3G degradation in MCE-CCP (50/50) (blu) and MCE-CCP (25/75) (orange) ingredients when exposed to a) 25 °C - 75% RH, b) 45 °C- 75% RH, c) 70 °C - 75% RH, d) 70 °C - 30% RH, and e) 80 °C - 30% RH. Degradation rates were extrapolated from the slope of the straight line (red line) going from the origin to the point corresponding to the specification limit of degradation.
